# Contextual and Social Characteristics Relevant to Health and Well-Being in African American and White Adult Populations

**DOI:** 10.1093/geroni/igaa057.1918

**Published:** 2020-12-16

**Authors:** Angela Sardina, Adrienne Aiken-Morgan, Alyssa Gamaldo

## Abstract

With the burgeoning older adult population, there will be an increased demand for neighborhood and housing developments conducive to the interests and needs of older adults from diverse backgrounds of varying health and functional status. Several initiatives have sought to develop age-friendly neighborhoods, which focused on improving access and affordability of community resources. However, limited effort has focused on physical and social attributes of immediate housing environments, particularly amongst lower-income older adults. The need for affordable and usable housing developments for older adults that provide greater opportunities for social engagement, social services, and convenience to neighborhood resources (e.g., grocery stores, healthcare) will continue to rise. The objectives of the proposed symposium are the following: (1) to explore the physical and social attributes of older and low-income residents’ housing and their surrounding community; and (2) discuss how older and low-income residents’ housing and community resources relates to their health and well-being. This symposium will include presentations from three pilot investigations that highlight relevant subjective and objective contextual metrics related to health and well-being in underserved older populations. Tan and colleagues explored the role of well-being (i.e., purpose in life) in the relationships among sociodemographics, health, housing and community resources. Sardina and colleagues explored perceived leisure barriers and their relationship to sociodemographic, health, and psychosocial characteristics. Aiken-Morgan and colleagues examined associations between neighborhood socioeconomic disadvantage and health status among low-income African American older adults. Wright and colleagues explored associations between neighborhood disadvantage, brain health, and neurocognitive function in cognitively normal older adults.

